# Hospital Based Emergency Department Visits Attributed to Child Physical Abuse in United States: Predictors of In-Hospital Mortality

**DOI:** 10.1371/journal.pone.0100110

**Published:** 2014-06-11

**Authors:** Veerajalandhar Allareddy, Rahimullah Asad, Min Kyeong Lee, Romesh P. Nalliah, Sankeerth Rampa, David G. Speicher, Alexandre T. Rotta, Veerasathpurush Allareddy

**Affiliations:** 1 Department of Pediatric Critical Care, Rainbow Babies and Children’s Hospital, Case Western Reserve University School of Medicine, Cleveland, Ohio, United States of America; 2 Department of Dental Medicine, Harvard University, Boston, Massachusetts, United States of America; 3 Department of Public Health, Texas A & M University, College Station, Texas, United States of America; 4 Department of Dentistry, University of Iowa, Iowa City, Iowa, United States of America; The University of Queensland, Australia

## Abstract

**Objectives:**

To describe nationally representative outcomes of physical abuse injuries in children necessitating Emergency Department (ED) visits in United States. The impact of various injuries on mortality is examined. We hypothesize that physical abuse resulting in intracranial injuries are associated with worse outcome.

**Materials and Methods:**

We performed a retrospective analysis of the Nationwide Emergency Department Sample (NEDS), the largest all payer hospital based ED database, for the years 2008–2010. All ED visits and subsequent hospitalizations with a diagnosis of “Child physical abuse” (Battered baby or child syndrome) due to various injuries were identified using ICD-9-CM (International Classification of Diseases, 9th Revision, Clinical Modification) codes. In addition, we also examined the prevalence of sexual abuse in this cohort. A multivariable logistic regression model was used to examine the association between mortality and types of injuries after adjusting for a multitude of patient and hospital level factors.

**Results:**

Of the 16897 ED visits that were attributed to child physical abuse, 5182 (30.7%) required hospitalization. Hospitalized children were younger than those released treated and released from the ED (1.9 years vs. 6.4 years). Male or female partner of the child’s parent/guardian accounted for >45% of perpetrators. Common injuries in hospitalized children include- any fractures (63.5%), intracranial injuries (32.3%) and crushing/internal injuries (9.1%). Death occurred in 246 patients (13 in ED and 233 following hospitalization). Amongst the 16897 ED visits, 1.3% also had sexual abuse. Multivariable analyses revealed each 1 year increase in age was associated with a lower odds of mortality (OR = 0.88, 95% CI = 0.81–0.96, p<0.0001). Females (OR = 2.39, 1.07–5.34, p = 0.03), those with intracranial injuries (OR = 65.24, 27.57–154.41, p<0.0001), or crushing/internal injury (OR = 4.98, 2.24–11.07, p<0.0001) had higher odds of mortality compared to their male counterparts.

**Conclusions:**

In this large cohort of physically abused children, younger age, females and intracranial or crushing/internal injuries were independent predictors of mortality. Identification of high risk cohorts in the ED may enable strengthening of existing screening programs and optimization of outcomes.

## Introduction

Child maltreatment occurs worldwide and is an important cause of morbidity and mortality. [1], [10] In United States, child maltreatment includes any act which results in physical, emotional or sexual abuse; or failure to act (neglect) resulting in imminent risk of harm, usually by a parent, caregiver or, occasionally, by an unknown perpetrator [2], [3].

Available estimates indicate that approximately 700,000 to 1.25 million children are abused or neglected annually in the United States [4], [5], [6] and about 18 percent of these cases involve physical abuse [7]. An estimated1.3% to 15% of injuries in children that result in emergency department visits are actually caused by physical abuse [8]. Physical abuse is often an underreported problem due to a variety of reasons including misdiagnosis. In fact, a study revealed that 31% of infants and children with abusive head trauma were initially misdiagnosed [9].

Although early detection rate of child abuse in emergency departments varies among different countries (Netherlands: 0.2%, Italy 2%, the United Kingdom: 4%–6.4%, United States: 10%) [16], [17], [18], [19], [20], [21] due to varied screening tools, emergency departments are important in the initial evaluation of suspected physical abuse in children [11], [12], [13], [14], [15]. Current national estimates and outcomes of physical abuse in children necessitating emergency department visit in United States are unclear.

The objective of our study is to describe nationally representative outcomes of physical abuse injuries in children necessitating hospital based emergency department (ED) visits in United States. The impact of facial and intracranial injuries on hospital mortality is also examined. We hypothesize that those with intracranial injuries are more likely to have higher in-hospital mortality compared to their counterparts.

## Materials and Methods

### Design, Database Description, Institutional Review Board and Data User Agreement

We performed a retrospective analysis of the Nationwide Emergency Department Sample (NEDS) for the years 2008 to 2010. The NEDS is a component database of the Healthcare Cost and Utilization Project (HCUP) sponsored by the Agency for Healthcare Research and Quality (AHRQ). [22] The NEDS is the largest all payer, nationally representative hospital based emergency department database in the United States. NEDS contains discharge data for ED visits from over 950 hospitals located in 30 States, approximating a 20-percent stratified sample of U.S. hospital-based EDs. The NEDS database has information on close to 100 different patient and hospital-related variables for each ED visit. Demographic data such as hospital and patient characteristics, geographic area, and the nature of ED visits (e.g., common reasons for ED visits, including injuries); and ED charge information for over 85 percent of patients, including individuals covered by Medicare, Medicaid, or private insurance, as well as those who are uninsured are available in NEDS [22].

As per University Hospitals Case Medical Center institutional review board (IRB) and in agreement with Federal Regulations 45 CFR 46.101 (b) which states “research involving the collection or study of existing data, documents, records, pathological specimens, or diagnostic specimens, if these sources are **publicly available** or if the information is recorded by the investigator in such a manner that **subjects cannot be identified**, directly or through identifiers linked to the subjects,” such studies are permitted to be classified as research that is “exempt” from IRB full or expedited review. IRB was not consulted for approval since the current study was a retrospective analysis of hospital based discharge dataset that is **available publicly** for purchase from AHRQ. The first author (VJA) completed the data user agreement with HCUP-AHRQ and the obtained the pertinent datasets. As per the data user agreement, cell counts ≤10 cannot be reported to maintain patient privacy. In accordance with the agreement, low cell counts are not reported and the term “DS” (Discharge information suppressed) is used instead.

### Case Selection and Outcome Variables Examined

All hospital based ED visits with a diagnosis of ***child physical abuse*** were selected for analysis. The ICD-9-CM code of 995.54 (battered baby or child syndrome) was used to identify these cases. In addition, in this cohort we also examined if patients had sexual abuse. This was identified by using the ICD-9-CM code of 995.53. Injury types were classified by using Clinical Classification Software codes and included Joint disorders and dislocations due to trauma (Clinical Classification Software code 225), fracture of neck of femur [hip] (226), spinal cord injury (227), skull and facial fractures (228), fracture of upper limb (229), fracture of lower limb (230), other fractures (231), sprains and strains (232), intracranial injury (233), crushing injury or internal injury (234), open wounds of head, neck, and trunk (235), and open wounds of extremities (236). Perpetrators of physical abuse were identified by using external cause of injury codes. The primary outcome variable of interest included hospital mortality (either in the emergency department or following hospitalization). The primary independent variables of interest included types of injuries.

The income quartiles varied by year. For 2008, the levels were $1–$38,999 (quartile 1), $39,000–$48,999 (quartile 2), $49,000–$63,999 (quartile 3), and > = $64,000 (quartile 4). For 2009, the levels were $1–$39,999 (quartile 1), $40,000–$49,999 (quartile 2), $50,000–$65,999 (quartile 3), and > = $66,000 (quartile 4). For 2010 the levels were $1–$40,999 (quartile 1), $41,000–$50,999 (quartile 2), $51,000–$66,999 (quartile 3), and > = $67,000 (quartile 4).

### Analytical Approach

A multivariable logistic regression model was used to examine the association between hospital mortality and types of injuries. Injuries that occurred in at least 1% of the ED visits were examined. The effects of age, sex, insurance status, and hospital region were adjusted in the multivariable logistic regression model. Odds ratios for hospital mortality and the associated 95% confidence intervals were computed. Each individual ED visit was the unit of analysis and the hospital stratum was the stratification unit. The effect of clustering of outcomes within hospitals was adjusted in the regression model. All statistical tests were two-sided a p-value of <0.05 was deemed to be statistically significant. Statistical analyses were conducted using SAS version 9.3 (SAS Institute, Cary, NC) and SUDAAN version 10.0.1 (Research Triangle Park, NC).

## Results

**Figure 1:**
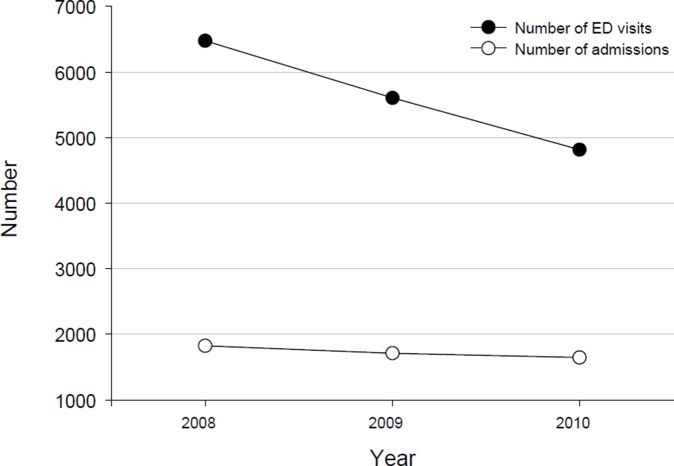
The number of ED visits and the number of hospitalizations due to physical abuse decreased over the analyzed time period (2008 to 2010).

During the study period, there were a total of 16,897 ED visits due to physical abuse against children-refer to table 1. Amongst these, 1.3% also reported a sexual abuse. Characteristics of the sample are summarized in Table 1. Medicaid (58.9%) and private insurance plans (23.8%) were primary payers for most ED visits, while 14.1% of patients were uninsured. Most patients (71%) were found to reside in geographic areas with low annual household income levels The number of ED visits and the number of hospitalizations due to physical abuse decreased over the analyzed time period (Figure 1).

**Table 1:** Characteristics of ED Visits due to Child Physical Abuse (Total N = 16,897).

Characteristic	Response	% of ED Visits
Sex	Male	55.9%
	Female	44.1%
Insurance status	Medicare	0.1%
	Medicaid	58.9%
	Private insurance	23.8%
	Uninsured	14.1%
	Other insurance	3.1%
ED visit	Weekday	75.6%
	Weekend	23.4%
Income quartile based on zip code*	Quartile 1	41.6%
	Quartile 2	29.4%
	Quartile 3	18.7%
	Quartile 4	10.3%
Hospital region	Northeast	24.5%
	Midwest	28.5%
	South	28.8%
	West	18.2%
Age in years	Mean	6.4 years
	Standard error	0.34

*Income quartile varied per year (see methods).

The types of injuries identified during the ED visit or subsequent hospitalization are summarized in Table 2, with intracranial injuries being the most prevalent. Commonly reported perpetrators of physical abuse included male partner of child’s parent/guardian (28.5% of all ED visits) and female partner of child’s parent/guardian (16.7%), unspecified person (14.4%) (Table 3).

**Table 2:** Types of Injuries in patients visiting the Emergency Department.

Type of injury(CCS Code)	% of all EDvisits(N = 16,897)	% of EDvisits inyear 2008(N = 6,477)	% of EDvisits inyear 2009(N = 5,605)	% of EDvisits inyear 2010(N = 4,815)
Joint disorders and dislocations; trauma-related (225)	0.2%	0.3%	DS	0.3%
Fracture of neck of femur [hip] (226)	0.4%	0.4%	0.3%	0.4%
Spinal cord injury (227)	DS	DS	DS	DS
Skull and facial fractures (228)	3.4%	2.9%	3.7%	3.7%
Fracture of upper limb (229)	6.3%	6%	6.4%	6.6%
Fracture of lower limb (230)	6.8%	6.7%	6%	7.9%
Other fractures (231)	5.4%	5.6%	4.8%	5.7%
Sprains and strains (232)	1.4%	1.4%	0.9%	2.1%
Intracranial injury (233)	11%	10.6%	10.5%	12%
Crushing injury or internal injury (234)	3%	2.8%	1.9%	4.7%
Open wounds of head, neck, and trunk (235)	5.6%	4.5%	6.7%	5.6%
Open wounds of extremities (236)	1.6%	1.5%	1.8%	1.6%

**Table 3:** Perpetrators of Physical Abuse.

Perpetrator	% of ED Visits*
Male partner of child’s parent or guardian	28.5%
Other specified person	5.5%
Female partner of child’s parent or guardian	16.7%
Abuse of spouse or partner by ex-spouse or ex-partner	0.4%
Child	0.4%
Sibling	1.4%
Grand parent	1.4%
Other relative	2.4%
Non-related care giver	1.4%
Unspecified person	14.4%

*Values do not add up to 100% due to missing or unreported data.

Following an ED visit, 65.2% of patients were discharged routinely, 2.5% were transferred to another short term hospital, 0.7% to long term care facilities like skilled nursing facility, 0.2% to home health care, and 0.3% were discharged against medical advice. A total of 5,182 ED visits (30.7%) resulted in admission as in-patients into the same hospital. A total of 246 patients died in hospitals (13 patients died in the ED and 233 died following in-patient admission). 0.3% were discharged-transferred to court/law enforcement agency.

**Figure 2:**
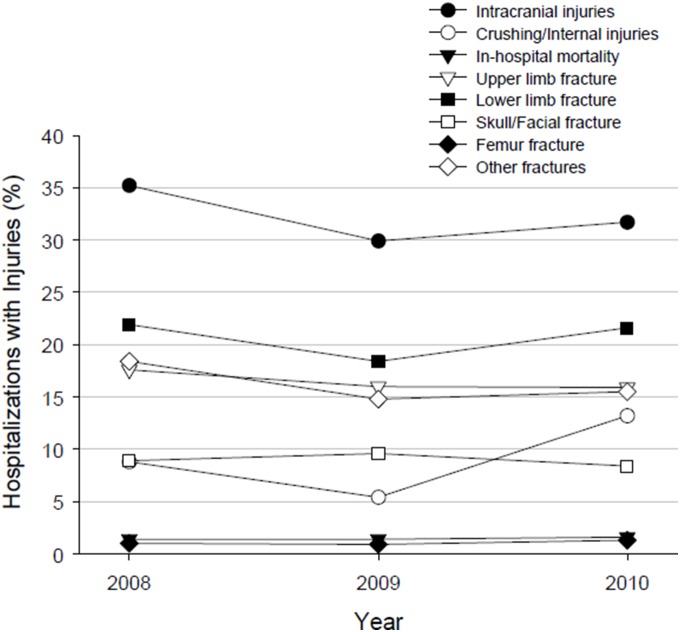
Common types of Injuries in Hospitalized Children.

**Figure 3:**
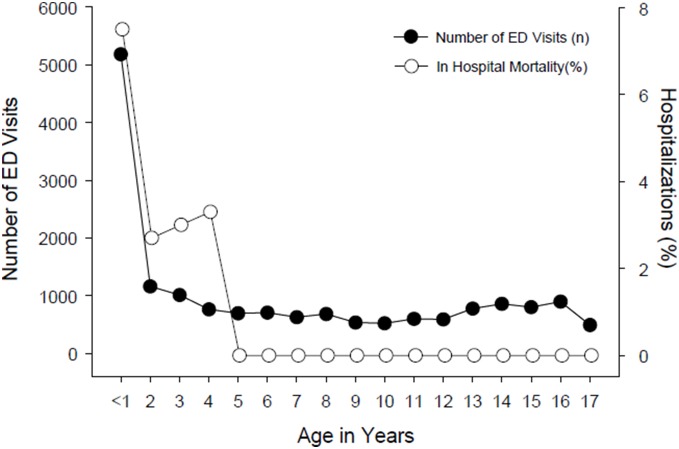
Effect of age on ED visits and in-hospital mortality. ED visits represented by left y axis and in-hospital mortality (%) by right y axis.

Characteristics of hospitalizations (ED visits that resulted in in-patient admission into the same hospital) are summarized in table 4. The mean age of hospitalizations was 1.9 years, which is lower compared to 6.4 years for those visiting the ED. Males comprised 61.3% of hospitalizations. Medicaid was the primary payer for 77.3% of hospitalizations. Close to 64.7% of hospitalizations occurred among the low income quartiles. Frequently occurring injuries among those hospitalized (Table 5) included intracranial injuries (32.3% of hospitalizations), fracture of lower limb (20.7%), and fracture of upper limb (16.5%). Skull and facial fractures occurred in 9% of hospitalizations. Figure 2 illustrates the common types of injuries in hospitalized children on a yearly basis. Intracranial injures were present in more than 30% of hospitalizations/year. Any type of fracture was present in >65% of hospitalizations/year and crushing/internal injuries were present in 5.4% to 13.2% hospitalizations/year. The in-hospital mortality was consistently 1.4 to 1.6% of hospitalizations/year. Figure 3 illustrates the effect of age on ED visits and in-hospital mortality. Younger age group children were more likely to visit the ED due to physical injury and had higher in-hospital mortality than their counterparts. Infants (≤1 year) had the highest in-hospital mortality rate of 7.5%.

**Table 4:** Characteristics of Hospitalizations (admitted as inpatient into same hospital following ED visit) [N = 5,182).

Characteristic	Response	% of Hospitalizations
Sex	Male	61.3%
	Female	38.7%
Insurance status	Medicare	0.2%
	Medicaid	77.3%
	Private insurance	15.8%
	Uninsured	3%
	Other insurance	3.7%
Admission	Weekday	75.5%
	Weekend	24.5%
Income quartile based on zip code*	Quartile 1	33.3%
	Quartile 2	31.4%
	Quartile 3	21%
	Quartile 4	14.3%
Age in years	Mean	1.9 years
	Standard error	0.20

*Income quartiles varied per year (see methods).

**Table 5:** Types of Injuries among those hospitalized.

Type of injury(CCS Code)	% of allHospitalizations(N = 5,182)	% ofHospitalizationsin year 2008(N = 1,826)	% ofHospitalizationsin year 2009(N = 1,710)	% ofHospitalizationsin year 2010(N = 1,646)
Joint disorders anddislocations; trauma-related (225)	0.5%	0.8%	DS	0.6%
Fracture of neck offemur [hip] (226)	1.1%	1%	0.9%	1.3%
Spinal cord injury(227)	DS	DS	DS	DS
Skull and facialfractures (228)	9%	8.9%	9.6%	8.4%
Fracture of upperlimb (229)	16.5%	17.6%	16%	15.9%
Fracture of lowerlimb (230)	20.7%	21.9%	18.4%	21.6%
Other fractures(231)	16.3%	18.4%	14.8%	15.5%
Sprains and strains(232)	0.5%	DS	DS	1.4%
Intracranial injury(233)	32.3%	35.2%	29.9%	31.7%
Crushing injury orinternal injury (234)	9.1%	8.8%	5.4%	13.2%
Open wounds ofhead, neck, andtrunk (235)	8.8%	9%	9.8%	7.8%
Open wounds ofextremities (236)	2.2%	1.2%	3.9%	1.6%

Results of the multivariable analyses examining the odds of hospital mortality (n = 246 deaths) are summarized in table 6. Each 1 year increase in age was associated with lower odds for mortality. Females were associated with higher odds for mortality compared to males. Patients with intracranial and those with crushing injury or internal injury had higher odds of mortality compared to those without these respective injuries. Hospitals located in the Southern regions of the country had lower odds for mortality compared to those located in the western regions.

**Table 6:** Characteristics associated with hospital mortality (multivariable logistic regression analysis).

Characteristic	Odds Ratio (95% CI)	p-value
Age (1 year increase)	0.88 (0.81–0.96)	<0.0001
**Sex**		
Female	2.39 (1.07–5.34)	0.03
Male	Reference	
**Insurance status**		
Medicaid	0.89 (0.41–1.92)	0.76
Medicare, private, uninsured, others	Reference	
**Type of injury**		
Skull and facial fractures	1.00 (0.18–5.55)	0.99
Intracranial injury	65.24 (27.57–154.41)	<0.0001
Open wounds of head, neck, and trunk	2.56 (0.67–9.73)	0.17
Fracture of upper limb	1.22 (0.58–2.55)	0.60
Fracture of lower limb	0.35 (0.10–1.27)	0.11
Other fractures	0.59 (0.24–1.41)	0.23
Sprains and strains	2.77 (0.40–19.12)	0.30
Crushing injury or internal injury	4.98 (2.24–11.07)	<0.0001
**Hospital region**		
Northeast	0.61 (0.27–1.39)	0.24
Midwest	0.73 (0.33–1.59)	0.42
South	0.41 (0.18–0.93)	0.03
West	Reference	

## Discussion

Child physical abuse affects all ages, genders, races, ethnicities and socioeconomic groups. [1], [3], [10]. To our knowledge this study is the largest and most recent cohort of children visiting the emergency department due to physical abuse whose risk of in-hospital mortality was assessed using a multitude of patient and hospital level characteristics at a national level. Using a large all payer national emergency department dataset, we show that younger age group, female gender, and intracranial or crushing/internal injuries are independent predictors of in-hospital mortality in those children admitted from the emergency department due to physical abuse.

In the present study nearly two-thirds of the patients presenting with physical abuse were routinely discharged from the ED. It is likely that these physical abuse injuries were in the less severe end of the spectrum. Such injuries could include bruises and abrasions due to slapping, beating or kicking. In addition, minor burns are a common cause of physical abuse in children. Although, any form of physical abuse is a concern and may require mandatory reporting to authorities in certain countries, in this study we sought to specifically describe the outcomes of physically abused children needing hospitalization. Hospitalized children are likely to have multiple injuries, higher severity of injuries or ongoing risk of exposure to perpetrator which requires hospitalization. Identification of certain types of injuries in high risk population groups in the emergency department may enable optimization of outcomes.

In our study, although children in the older age group were likely to visit the ED for physical abuse, children in the younger age group were more likely to be admitted and the risk of in-hospital mortality significantly decreased with increasing age. This is consistent with prior findings that although the risk of physical abuse increases with age [34], fatal abuse is more common among infant and children younger than 2 years [35]. Infants had the highest risk of mortality. Possible explanations include higher severity of injuries, delay in seeking medical attention due to non-specific symptoms/signs, repeated abuse by perpetrator over a period of time before suspicion is confirmed, or multiple injuries that infants are at risk for (intracranial, abdominal organ lacerations, fractures). Also, older age group children are more likely to report and seek medical attention for physical abuse than younger age group children.

Further, in our study, males were more likely to visit ED and get admitted for physical abuse; however, females were associated with significantly higher risk of in-hospital mortality. This gender difference may be due to presence of other concomitant injuries such as sexual abuse in female children [40]. However, this premise merits empirical support through future studies. In the present study, amongst the 16897 ED visits 1.3% had associated sexual abuse. A prior study had revealed that children living in lower annual income households (<$15000 per year) had 3 times the number of fatalities, 7 times the number of serious inflicted injuries, and 5 times the number of moderate inflicted injuries when compared to their counterparts [36]. In our study, 70% of children who visited ED and more than 60% of those who were admitted following a physical abuse injury were from homes with lower income quartiles. Further studies are needed to explore the complex relationship between income levels and child abuse. In our study, the type of insurance did not influence the risk of mortality.

Head injury is the leading cause of child abuse fatalities [33]. In our study, abused children with intracranial injury or crushing/internal injury had a significantly higher risk of in-hospital mortality. This is consistent with prior studies which showed that infants or children with head or abdominal injuries due to physical abuse are more likely to die or become severely debilitated than are children with accidental head or abdominal injuries. [30], [31], [32], [39]. In our study, although, more than 60% of hospitalized children with physical abuse had some form of fractures, their presence did not influence the in-hospital mortality. A significant association for increased risk of abusive head trauma by non-parental perpetrator in the older children was identified in a prior study [40]. In our study, male or female partner of child’s parent or guardian was the most common perpetrator of physical abuse, which is consistent with prior findings [37].

There are methodological limitations to our study pertaining to the use of large administrative datasets. Although, the retrospective nature of the study precludes us from drawing a definite cause and effect relationship between the independent variables and occurrence of a specific event, we have shown associations between the type of physical abuse injury and outcomes which are consistent with prior research. In addition, though we used a multivariable logistic regression analysis to account for the confounding effects of patient- and hospital-level variables, the risk adjustment performed is not comprehensive due to the lack of adequate patient level clinical data (e.g. PRISM scores, GCS scores, retinal hemorrhages, intraparenchymal hemorrhage, and cerebral edema) in the NEDS dataset that have been shown to influence outcomes [39]. The use of ICD-9-CM codes for identification of physical child abuse cases may be prone to coding and or billing underestimates [24], [25], [26]. However, a recent study demonstrated the effective use of ICD-9-CM codes for child maltreatment conditions [23]. In addition, a recent study demonstrated very high specificity in identifying child physical abuse using ICD-9-CM codes. [24]. Our study is limited to children with injuries due to physical abuse visiting the emergency departments and hence does not capture hospitalizations from the primary care provider or inter-hospital transfers without emergency department triage. Victims of physical abuse are more likely to develop significant long-term medical and psycho-social morbidity [27], [28], [29], [38]. The nature of the dataset precludes us from further evaluation of outcomes following discharge. Neglect or abuse due to sexual or emotional causes were not evaluated in this study and should be the focus in future studies.

Despite these limitations, our study findings are likely representative of practices beyond single center experiences, and hence generalizable. Identification of high risk cohorts in emergency department may enable strengthening of existing screening programs and optimization of outcomes. Given the unique position emergency care professionals hold in the health care system, opportunities exist for them to become a major stakeholder in the care of physically abused children.

## Conclusion

Physical abuse is not an uncommon cause of emergency department visits in children in United States. In this large cohort of hospitalized children with physical abuse, younger age group, female gender and intracranial or crushing/internal injuries were independent predictors of in-hospital mortality.
